# Draft Genome Sequence of the Extremely Desiccation-Tolerant Cyanobacterium Gloeocapsopsis sp. Strain AAB1

**DOI:** 10.1128/genomeA.00216-18

**Published:** 2018-04-26

**Authors:** Fernando Puente-Sánchez, Carlos González-Silva, Victor Parro, Javier Tamames, Armando Azua-Bustos

**Affiliations:** a Systems Biology Program, Centro Nacional de Biotecnología, Consejo Superior de Investigaciones Científicas (CSIC), Madrid, Spain; b Centro de Investigación del Medio Ambiente (CENIMA), Universidad Arturo Prat, Iquique, Chile; c Centro de Astrobiología (CSIC-INTA), Madrid, Spain; d Instituto de Ciencias Biomédicas, Facultad de Ciencias de la Salud, Universidad Autónoma de Chile, Santiago, Chile

## Abstract

Gloeocapsopsis sp. strain AAB1 is an extremely desiccation-tolerant cyanobacterium isolated from translucent quartz stones from the Atacama Desert (Chile). Here, we report its draft genome sequence, which consists of 137 contigs with an ∼5.4-Mb genome size. The annotation revealed 5,641 coding DNA sequences, 38 tRNA genes, and 5 rRNA genes.

## GENOME ANNOUNCEMENT

Members of the Cyanobacteria are among the most ecophysiologically resilient microorganisms, and they have been extensively studied in order to understand the limits of life on Earth (1). Gloeocapsopsis sp. strain AAB1 is a cyanobacterium isolated from a hypolithic community found under translucent quartz stones dispersed on the Coastal Range of the Atacama Desert, the driest and oldest desert on Earth. Although rain events in the Atacama are rare, cyanobacterium-dominated biofilms are able to colonize the underside of quartz stones, using fog as the main source of water (2). The recent physiological, morphological, and molecular characterization of isolate AAB1 revealed that it is extremely tolerant to desiccation (3) and represents a good model for understanding tolerance to low water availability.

Although Gloeocapsopsis cells were regularly grown using liquid BG-11 media, for the purpose of sequencing, DNA was extracted from 5 mg of dried cells. We used dried cells because, although we were confident of having a single cyanobacterial isolate (3), we detected 3 or 4 additional heterotrophic bacteria that we could not eliminate despite attempting different cleaning protocols. Thus, cells were immediately dried, with the expectation that the desiccation process would favor the preferential preservation of cyanobacterial DNA. DNA was extracted using the Qiagen DNeasy PowerSoil kit (Qiagen, Venlo, Netherlands) according to the manufacturer’s instructions, except that at the cell lysis step only a single 120-s pulse was performed, using a FastPrep24 5G homogenizer (MP Biomedicals, Santa Ana, CA). The sequencing library was prepared using a NEBNext Ultra II DNA library prep kit for Illumina (New England Biolabs, Ipswich, MA), following the manufacturer’s instructions, and sequenced in a 2 × 300 pair-end sequencing run on a MiSeq sequencer (Illumina, San Diego, CA).

Raw reads were quality trimmed with Prinseq Lite v0.20.4 (4). The resulting 5,830,860 pairs were processed with the SqueezeM pipeline (J. Tamames, unpublished data). Briefly, they were assembled using MEGAHIT v1.1.2 (5), and open reading frames (ORFs) and rRNA coding sequences were predicted with Prodigal v2.6.2 (6) and barrnap v0.9-dev (https://github.com/tseemann/barrnap), respectively. ORFs were aligned against the Kyoto Encyclopedia of Genes and Genomes (KEGG) (7) and Clusters of Orthologous Groups of proteins (COG) (8) databases using DIAMOND (9), functionally annotated based on their best hits, and taxonomically annotated based on a last-common-ancestor algorithm over all hits within 20% of the bit score of the first hit. Furthermore, whole contigs were assigned a consensus taxonomy based on the annotation of their constituent ORFs. A total of 137 high-coverage contigs, encompassing a total of 5,443,570 bases, were identified as having a clear cyanobacterial origin and selected for further analyses. Among these contigs, the *N*_50_ value was 73,596 bases, the average coverage was 509×, and the GC content was 42.42%. CheckM v1.0.7 (10) reported a genome completeness of 100% and genome contamination of 1.36%, after being run on the cyanobacterial contigs. Finally, gene prediction and annotation of the Gloeocapsopsis sp. AAB1 contigs were carried out using the Rapid Annotations using Subsystems Technology (RAST) pipeline (11) as provided by the Pathosystems Resource Integration Center (PATRIC) server (12). A total of 5,641 coding DNA sequences (CDSs), 38 tRNA genes, and 5 rRNA genes were identified.

### Accession number(s).

The nucleotide sequence and annotation data for the Gloeocapsopsis sp. strain AAB1 draft genome have been deposited at DDBJ/ENA/GenBank under the accession number PTJN00000000. The version described in this paper is version PTJN01000000.
